# Plasma Lipidomics of Preadolescent Children: A Hokkaido Study

**DOI:** 10.1155/jl/3106145

**Published:** 2025-02-16

**Authors:** Jayashankar Jayaprakash, Siddabasave Gowda B. Gowda, Divyavani Gowda, Atsuko Ikeda, Yu Ait Bamai, Rahel Mesfin Ketema, Reiko Kishi, Yifan Chen, Hitoshi Chiba, Shu-Ping Hui

**Affiliations:** ^1^Graduate School of Global Food Resources, Hokkaido University, Kita-9, Nishi-9, Kita-Ku, Sapporo, Japan; ^2^Faculty of Health Sciences, Hokkaido University, Kita-12, Nishi-5, Kita-ku, Sapporo, Japan; ^3^Center for Environmental and Health Sciences, Hokkaido University, Kita-12, Nishi-7, Kita-ku, Sapporo, Japan; ^4^Department of Nutrition, Sapporo University of Health Sciences, Nakanuma Nishi-4-2-1-15, Higashi-ku, Sapporo, Japan

**Keywords:** childhood obesity, children, lipidomics, liquid chromatography, mass spectrometry, plasma

## Abstract

Lipids are the most abundant biomolecules of human plasma, and their balance plays a significant role in health and disease management. Despite the importance of lipids, the studies focused on the comprehensive determination of the plasma lipidome in children are limited. In this study, we investigated the sex, age, and weight-specific changes in the plasma lipidome of nonfasting preadolescent children aged 9–12 years (*n* = 342) using a nontargeted liquid chromatography−mass spectrometry technique. A total of 219 lipid species were characterized in the plasma samples. Multivariate analysis revealed that boys and girls have similar lipid profiles, but relatively higher levels of capric acid–composed triacylglycerols (TGs) were observed in plasma samples of boys. Saturated fatty acids are the most abundant fatty acyls followed by mono- and polyunsaturated fatty acids in the plasma of both boys and girls. Sphingolipids such as ceramides, hexosylceramides, sphingomyelin, and a phospholipid (phosphatidylinositol) were relatively higher in the plasma of a 10-year-old group than other age groups. Plasma levels of TG and phosphatidylserine were increased within age from 9 to 12 years. Furthermore, most of the TG molecular species were increased in the plasma of overweight children compared to the normal range groups. The receiver operating characteristic analysis results show that TG (10:0/10:0/18:1) could be a specific marker for childhood obesity (area under the curve (AUC) = 0.72). Overall, this study highlights the altered plasma lipidome in preadolescent children for sex, age, and percentage of overweight. Early detection of lipid markers for obesity would be a promising target for developing therapeutic strategies.

## 1. Introduction

Lipids are crucial molecules in human health, including cellular and extracellular components, and significantly contribute to cell structure, function, energy storage, body insulation, and protection [1]. Lipid metabolites are important mediators in the human body's cellular communication and metabolic processes. Dysregulated lipid metabolism is associated with a spectrum of chronic diseases, including cardiovascular disease, cancer, and genetically influenced lipid pathway disorders, which have severe health implications for humans [2]. During early childhood, lipid metabolism facilitates growth, establishes body composition, fosters child development, and influences long-term health outcomes [3]. Dietary lipids influence cholesterol metabolism during early childhood, potentially affecting the health outcomes of children, including morbidity and mortality [4]. To evaluate disease risk, plasma lipids are regularly assessed using profiling of total cholesterol, triacylglycerols (TGs), high-density lipoprotein cholesterol (HDL-C), and low-density lipoprotein cholesterol (LDL-C) [5].

Human plasma contains thousands of molecular lipid species that are functionally and chemically diverse in addition to nucleic acids, amino acids, and carbohydrates [6]. Plasma lipids consist of micrometers to millimeters concentrations of various lipid categories such as fatty acyls (FAs), glycerolipids (GLs), glycerophospholipids (GPs), sphingolipids (SPs), sterols (STs), and prenols [7]. Lipid metabolites in human plasma are linked to various biological processes and serve as biomarkers, reflecting disease states and responses to therapeutic drugs [8]. Total TG, HDL-C, and LDL-C concentrations in plasma are significant indicators and potential causes of future risk for cardiometabolic diseases [9]. Thus, these lipoprotein biomarkers are routinely examined in clinical decision-making and the associated molecular pathways are key targets of first-line drugs for prevention of cardiometabolic diseases [10]. Furthermore, various plasma lipid classes such as TG, GPs, SPs, and cholesterol esters (CEs), have been reported as diagnostic markers of Alzheimer's disease [11], lysophosphatidylcholine (LPC) as a risk factor for cancers [12], and SPs as useful parameters for monitoring the effectiveness of therapeutic drugs for hereditary illnesses such as the Niemann–Pick C1 disease [13]. The interplay of diet, physical activity, and genetic factors influences the altered lipid metabolism in human plasma samples [14, 15]. In this line, previous reports observed that dietary and physical activity interventions positively impact fatty acid composition in plasma samples of 6- to 8-year-old children [16]. The study by Martin et al. showed the upregulation of LPC species in the plasma of obese women after exercise [17]. Another study by Zhang et al. reported that systematic exercise training led to increased levels of ether-linked alkylphosphatidylcholine and TG levels in the plasma of healthy male adolescents [18]. Furthermore, genetic variation also plays a pivotal role in shaping the human plasma lipidome with distinct loci linked to specific lipid species that are associated with cardiovascular disease risk [19].

Past studies on plasma lipidome in children are mostly focused on a specific class of lipids. A recent cohort study analyzed plasma samples from healthy adolescents to measure total cholesterol, HDL-C, and TG levels using a standard enzymatic method. The results indicated that total cholesterol levels were decreased from ages 9 to 16 years in girls and more steeply from ages 10 to 17 years in boys. Further, the levels of TG increased from ages 8 to 12 years among girls and a linear change from ages 8 to 18 years among boys [20]. However, liquid chromatography–mass spectrometry (LC/MS) is a widely applied technique for plasma lipidomics. For example, lipids from dried blood spots collected from children of different age groups (0–10 days, 2–18 months, and 3–13 years) were extracted using the Bligh-Dyer method and analyzed using the LC/MS. The analysis results revealed that 16 distinct lipid species from different subclasses of lipids show statistically significant differences across the different age groups. Additionally, lipid species containing linoleic acid were significantly lower in the 0–10 days group with a gradual increase from 2–18 months and 3–13-year group of children [21]. LC/MS technique was also applied to profile the plasma lipidome of Chinese obese adolescents and normal-weight groups of male subjects of the 14–16-year age group. Lipids from plasma samples were extracted using a modified Folch technique, and the results showed that 328 lipid species from 24 lipid classes and subclasses were identified. They found a significant reduction in plasma LPCs of obese-preadolescents compared to normal-weight subjects and showed a strong association between LPCs with adolescent obesity [22].

**Figure 1:**
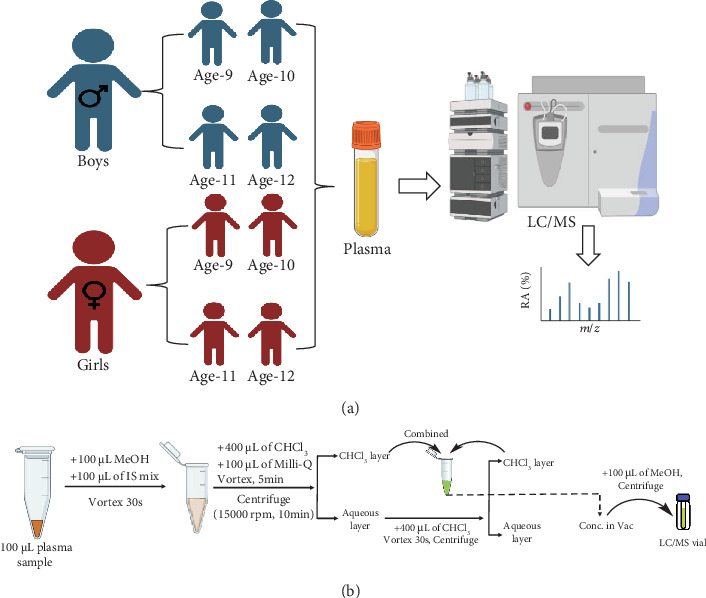
(a) Study design strategy for plasma sample analysis of preadolescent children. (b) Workflow for total lipid extraction from plasma samples.

Existing studies on plasma lipidomics in healthy children are limited in understanding how age, sex, and body weight can influence lipid metabolism and associated lifestyle-related diseases. To the best of our knowledge, there have been no extensive lipid profiling studies conducted on Japanese preadolescents. It is of great interest to explore the plasma lipidome of Japanese children and their variations with age, sex, and body weight. Hence, in this study, we used high-performance liquid chromatography in combination with linear-ion trap quadrupole-orbitrap mass spectrometry (HPLC/LTQ-Orbitrap-MS) to systematically investigate the comprehensive lipidome of the plasma samples obtained from boys and girls preadolescent children aged 9–12 years (*n* = 342). The detailed study design is depicted in Figure 1(a). To the best of our knowledge, this study is the first attempt to conduct a comprehensive lipid analysis of plasma samples from preadolescent Japanese children, considering different ages, sexes, and body weights.

## 2. Materials and Methods

### 2.1. Chemicals and Reagents

LC/MS-grade solvents, including methanol, isopropanol, and a mobile-phase additive, 1 M aqueous ammonium acetate, were obtained from Wako Pure Chemical Industries, Ltd. (Osaka, Japan). HPLC-grade chloroform was purchased from Sigma-Aldrich (Missouri, United States). The internal standard (IS) EquiSPLASH (Lot: 330731-1EA-013) and oleic acid-d9 were obtained from Avanti Polar Lipids (Alabaster, Alabama).

### 2.2. Preadolescent Children's Plasma Samples

Nonfasting plasma samples from individuals were provided by participants in the Hokkaido Study on Environment and Children's Health, part of the Hokkaido Cohort [23], and stored at −80°C. The selection criteria of participants for sample collection were previously reported in our study [24]. The study comprised 342 healthy preadolescent children (*n* = 181 boys and 161 girls) residing in Sapporo City, Hokkaido, Japan, between the ages of 9 and 12. The children's height and body weight were measured at pediatric clinics on the same day as blood collection. The detailed demographics of preadolescent children are provided in Table 1. All parents provided written informed consent, and all children provided informed consent, after being educated about the study's goals and procedures.

### 2.3. Extraction of Lipids From Plasma Samples

Nonfasting plasma samples (*n* = 342) from preadolescent boys and girls aged 9–12 years old were subjected to total lipid extraction. The samples were collected and stored at −80°C for further analysis. Extraction of plasma samples was performed by using the modified Folch method established earlier in our laboratory [25, 26]. The detailed extraction workflow is shown in Figure 1(b). In brief, 100 *μ*L of plasma was taken into a 2 mL Eppendorf tube, followed by the addition of 100 *μ*L of methanol and 100 *μ*L of an IS solution. The IS solution consists of oleic acid-d9 (100 *μ*g/mL) and EquiSPLASH (10 *μ*g/mL) mixtures in methanol. Followed by the addition of 400 *μ*L of chloroform and 100 *μ*L of Milli-Q, the mixture was vigorously vortexed for 5 min and centrifuged at 15,000 rpm for 10 min to yield biphasic layers. The lower layer chloroform-containing extracted lipids was transferred to a new 2 mL Eppendorf tube. To the upper layer, another 400 *μ*L of chloroform was added, vortexed for 1 min, and centrifuged for 10 min under the conditions described above. Then, the second-time extracted chloroform layer was transferred to the same 2 mL Eppendorf tube. The combined chloroform extracts were evaporated in a centrifuge evaporator at 4°C for 3 h and then redissolved in 100 *μ*L of methanol. Before transferring to LC vials, the lipid extracts in methanol were centrifuged for 10 min and injected 10 *μ*L of the sample.

### 2.4. LC/MS Analysis of Plasma Samples

The LC/MS conditions were consistent with those employed in our previous studies [27]. LTQ Orbitrap XL MS (Thermo Fisher Scientific Inc., San Jose, California), coupled with HPLC using an LC-20AD UFLC system (Shimadzu Corp., Kyoto, Japan), was used for lipidomic analysis. An Atlantis T3 C18 column (2.1 mm × 150 mm, 3 *μ*m, Waters, Milford, Massachusetts) that was placed in a 40°C oven temperature was used for lipid separation with a mobile phase flow rate of 0.2 mL/min ((A) aqueous 10 mM ammonium acetate (CH_3_COONH_4_), (B) isopropanol, and (C) methanol). All the MS parameters were identical to those of our previous report [28, 29]. Analysis was conducted in both positive and negative ionization modes. Electrospray ionization (ESI) was used with the following conditions: the capillary temperature was set to 330°C, the nitrogen sheath gas flow was set to 50 units, and the nitrogen auxiliary gas flow was set to 20 units. Source and capillary voltages were adjusted to 3 kV and 10 V for negative ionization mode (scan range: *m*/*z* 160–1900) and 4 kV and 25 V for positive ionization mode (scan range: *m*/*z* 150–1950). Analysis was performed in Fourier transform mode with a resolving power of 60,000 to obtain MS spectra and in ion-trap mode to obtain MS/MS spectra at a collision energy of 40 V.

### 2.5. Lipid Annotation and Quantification

The raw data obtained from LC/MS was processed using MS-DIAL software Version 4.9, to perform tasks such as data alignment, peak extraction, identification, and integration of peak areas [30]. The Xcalibur 2.2 (Thermo Fisher Scientific, Waltham, United States) software was used to guarantee the mass spectra of reliable identification of lipid molecular species and manually verify the integrated peak areas. Relative quantification of lipid molecular species was performed by calculating peak area ratios of identified lipids to the added ISs. These area ratios were then multiplied by the concentration of the added IS and normalized to the sample volume to determine the relative amount of lipid in plasma samples.

### 2.6. Statistical Analysis

The dataset utilized in this study comprised lipid molecular species analyzed in both positive and negative ionization modes. Data visualization was conducted using Microsoft Excel 2021, and the results were plotted using GraphPad Prism Version 8.0.1. Statistical analyses including orthogonal partial least squares discriminant analysis (OPLS-DA) were performed using the web platform MetaboAnalyst 5.0 (https://www.metaboanalyst.ca, accessed on January 4, 2024), following the provided user instructions. Score plots were generated to assess similarity among different groups, and loading plots (model coefficients versus covariance) were utilized to identify discriminating features in lipid profiles for each group. G⁣^∗^Power Version 3.1.9.7 software was used to perform the power analysis. All data were presented as mean ± standard error of the mean (SEM).

## 3. Results

### 3.1. Multivariate Analysis and Sex-Specific Lipid Fingerprinting of Plasma Samples of Preadolescent Children

**Figure 2:**
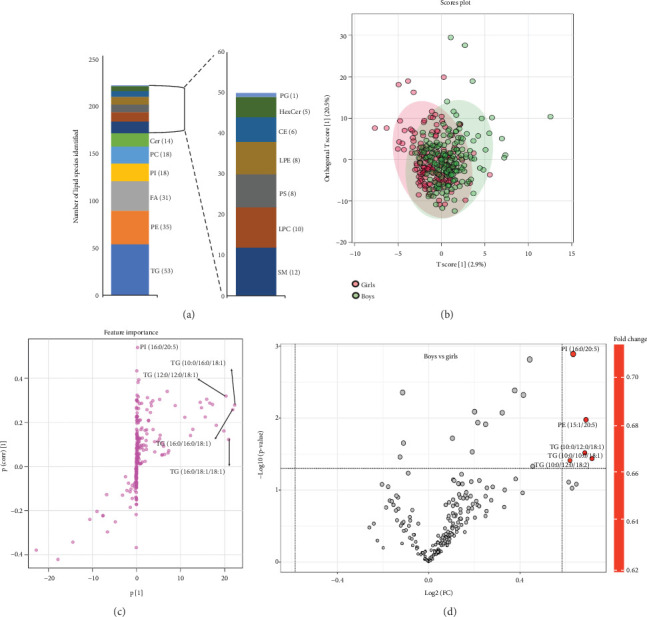
(a) Different classes of lipid species identified in preadolescent children's plasma samples. (b) Orthogonal partial least squares discriminant analysis (OPLS-DA) score plot of the plasma samples from the boy and girl group of preadolescent children's plasma samples. (c) Loading plot and scores of lipid species from both sexes. (d) Volcanic plot representing significantly altered lipid levels (*p* < 0.05) in the plasma samples of boy versus girl preadolescent children's group.

In this study, preadolescent children were categorized by gender, and plasma samples were collected for the total lipid extraction. Subsequently, nontargeted lipid analysis was conducted using HPLC/LTQ-Orbitrap-MS.  Figure 2 represents the different classes of lipid species identified in preadolescent children's plasma samples and their multivariate analysis. The list of identified lipid species and their relative concentrations in the plasma samples and physical exercise pattern of preadolescent children are provided in the Supporting Information Tables S1 and S2. Power calculations were performed to justify the adequacy of the sample size used in this study. The power analysis between children's groups based on body weight showed a power value of 0.99, suggesting a good justification for sample size between bodyweight groups. However, the power analysis between the boys and girls sample size group showed a power value of 0.15 which is very low, a poor justification was observed. The detailed power calculation values are provided in Table S3.

The analysis of plasma samples from preadolescent children revealed 219 lipid species spanning various lipid classes (Figure 2(a)). Among these, 53 were identified as TG, followed by 35 phosphatidylethanolamines (PEs), 31 FAs, 18 phosphatidylinositols (PIs), 18 phosphatidylcholines (PCs), and 14 ceramides (Cer). The multivariant analysis of the OPLS-DA score plot of the annotated lipids from both the boy's and girl's groups shows a similar lipid profile, as illustrated in Figure 2(b). The two models of *T* scores, *T* score 1 and *T* score 2, accounted for 23.4% of the total variance of the model, which was described by 20.5% of the *T* score 1. Score plots and their feature importance box plot (also known as S-plot) visualize the variable influence in the OPLS-DA model. Larger positive or negative loading scores indicate the influence of a variable on the orthogonal components.  Figure 2(c) shows that TG lipid molecular species have relatively higher positive loading scores, such as TG (10:0/16:0/18:1), TG (12:0/12:0/18:1), TG (16:0/16:0/18:1), and TG (16:0/18:1/18:1) in boys.

A volcanic plot represents the graph of −log_10_(*p* value) versus log_2_(fold change), describing the significantly and largely altered lipid molecular species between the boy's and girl's plasma lipidome (Figure 2(d)). The findings unequivocally demonstrate noteworthy higher levels of specific lipid molecules in the boys than in the girls. Notably, PE (16:0/20:5), PE (15:1/20:5), TG (10:0/12:0/18:1), TG (10:0/10:0/18:1), and TG (10:0/12:0/18:2), which are prominently highlighted in red on the right side of the plot, were higher in boys than in the girls. However, there were no discernible instances of downregulation among lipid molecules in the plasma between boys and girls.

The lipid biosynthesis pathway analysis in humans is depicted in Supporting Information 1: Figure S1 (based on Kyoto Encyclopedia of Genes and Genomes (KEGG) pathways (https://www.genome.jp/kegg/pathway.html)). Our lipidomic analysis shows the variations in specific lipid biosynthesis pathways between boy's and girl's plasma lipidomes. Notably, there is no significant difference in fatty acid levels has been observed between boys and girls. The hexosylceramides (HexCer) and sphingomyelin (SM) are decreased in girls compared to boys. This may be due to the downregulation of the SP biosynthesis pathway in girls. Similar observations have been observed in the case of GPs and GLs. These observations provide insights into sex-specific lipid metabolism and its regulation.

### 3.2. Variation in the Plasma FA Level in Preadolescent Children of Different Age Groups

**Figure 3:**
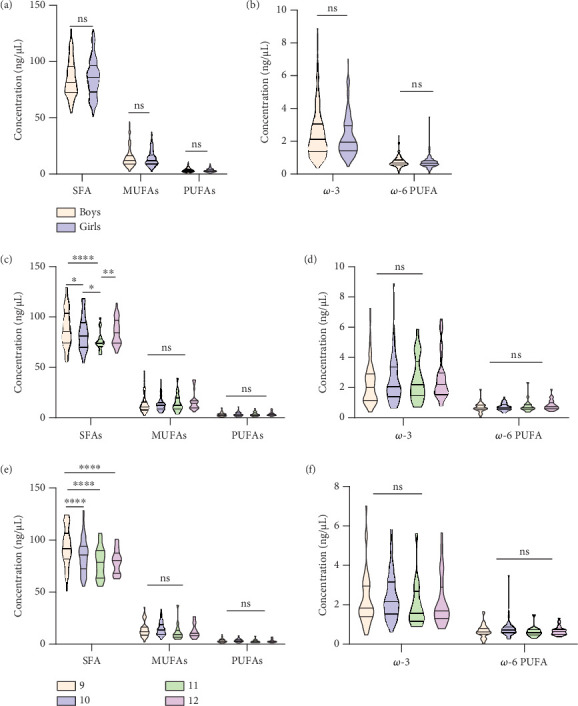
(a) Changes in SFA, MUFA, and PUFA levels in boys and girls. (b) *ω*-3 and *ω*-6 PUFA levels in boys and girls. (c) Changes in SFA, MUFA, and PUFA levels in boys with different age groups. (d) *ω*-3 and *ω*-6 PUFA levels in boys with different age groups. (e) Changes in SFA, MUFA, and PUFA levels in girls with different age groups. (f) *ω*-3 and *ω*-6 PUFA levels in girls with different age groups (ordinary two-way ANOVA, ⁣^∗∗∗∗^*p* < 0.0001, ⁣^∗∗^0.01, ⁣^∗^0.05, 0.1 (ns), saturated fatty acids (SFAs), monounsaturated fatty acids (MUFAs), and polyunsaturated fatty acids (PUFAs)).

Figure 3 depicts notable alterations in FA levels across various age groups in both boy's and girl's plasma lipidome subjects. Violin plots illustrate the average concentrations of saturated fatty acids (SFAs), monounsaturated fatty acids (MUFAs), and polyunsaturated fatty acids (PUFAs) (including *ω*-3 and *ω*-6 PUFAs) based on individual concentrations of molecular species within each group. Surprisingly, there were no statistically significant changes observed in the overall levels of SFAs, MUFAs, and PUFAs in both boy's and girl's groups (Figure 3(a)). Although PUFAs have a positive impact on metabolic health, no significant changes were observed in *ω*-3 and *ω*-6 PUFA levels in both sexes as shown in Figure 3(b). When examining different age groups (9, 10, 11, and 12 years), distinct variations in FA levels were observed. Specifically, a substantial increase in SFA levels was identified in the plasma of boys aged 9 years. However, the rate of increase was higher in the 9-year-old group than in the other age groups. In contrast, a distinctive pattern is also observed in boys of different ages. Specifically, the SFA level exhibited a significant decrease in the 11-year-old group.

Notably, no significant changes were observed in the levels of MUFAs and PUFAs (both *ω*-3 and *ω*-6 PUFAs) (Figures 3(c) and 3(d)). Similarly, among the girls, a significant increase in SFA levels was observed in the 9-year-old group compared with the other age groups. Remarkably, the rate of increase in SFA levels mirrored that of the boy group, with a notably higher rate in the 9-year-old girl group than in the other age groups. However, no significant changes were noted in SFAs, MUFAs, and PUFAs (both *ω*-3 and *ω*-6 PUFAs) levels among the different age groups of girls other than the 9-year-old group. The MUFAs and PUFA levels displayed no significant changes across different age groups among the girls (Figures 3(e) and 3(f)). SFA levels in the 9-year-old group, irrespective of sex, were higher than those in the other age groups. However, no significant changes were observed in MUFAs and PUFAs across different age groups in boys and girls.

### 3.3. Variation in the Plasma Lipidome at the Subclass Level in Preadolescent Children of Different Age Groups

**Figure 4:**
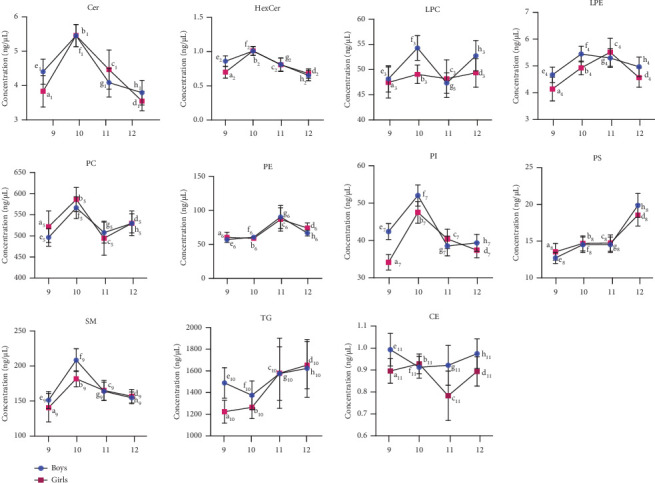
Change in plasma lipidome at subclass levels in the 9-, 10-, 11-, and 12-year age groups of both boys and girls. The variation of girls from different age groups is shown in red color, and boys with different age groups are shown in blue color (ordinary two-way ANOVA, *p* < 0.05 is considered statistically significant). The significant groups are shown as follows: Cer: girls (a1–b1, b1–d1), boys (f1–h1). HexCer: girls (a2–b2, b2–d2), boys (f2–h2). LPC: ns. LPE: ns. PC: ns. PE: girls (a6–c6, b6–c6), boys (e6–g6, f6–g6). PI: girls (a7–b7), boys (e7–f7, f7–g7, f7–h7). PS: girls (a8–d8), boys (e8–h8, f8–h8). SM: boys (e9–f9). TG: ns. CE: ns (*p* > 0.05 (ns)).

The lipid molecular species were grouped based on their subclass levels; the relative amounts of altered lipidomes in both sexes based on age are illustrated in Figure 4. The line graph shows the mean ± SEM of the individual concentrations of each molecular species in the corresponding groups, representing the concentration of each lipid subclass. SPs such as Cer, HexCer, and SM were significantly elevated in the 10-year age group of both sexes compared with the other age groups. GP lipids such as PE were, significantly increased in the 11-year age group in both boys and girls, but no significant changes were observed in the 9-, 10-, and 12-year-old groups of children in both sexes. Similarly, the PI lipid levels were significantly increased in the 10-year age group of both boys and girls compared with the other age groups of both sexes. Total phosphatidylserine (PS) lipids were elevated in the 12-year-old children but significantly lower in the 9-year-old group of both boy and girl children. However, there is no significant changes have been observed in the cases of LPC, lysophosphatidylethanolamine (LPE), and PC.

Furthermore, GL lipids, such as TG, were significantly elevated in the 12-year age group of both sexes compared with the other age groups. As shown in Figure 4, the 10- and 12-year age groups of both boys and girls showed significant variations in different subclasses of lipids. In conclusion, increased Cer, HexCer, SM, and PI are specific markers for the 10-year age group of children of both sexes. Elevated levels of TG and PS have emerged as potential markers for distinguishing between boys and girls in preadolescent children within the 12-year age group. Supporting Information 1: Figures S2 and S3 show volcano plots, illustrating the relationship between −log_10_(*p* value) and log_2_(fold change) to elucidate significantly altered lipid molecular species within the boy's and girl's plasma lipidomes across different age groups. Notably, in the 10-year-old group of children, there is a significant upregulation of Cer (d18:1/23:1) in both boys and girls. However, HexCer (d18:1/16:0) appeared to decrease in the 11 and 12-year age groups of children of both sexes.

### 3.4. Percentage of Overweight (POW) Effect on Plasma Lipidome of Preadolescent Children

**Figure 5:**
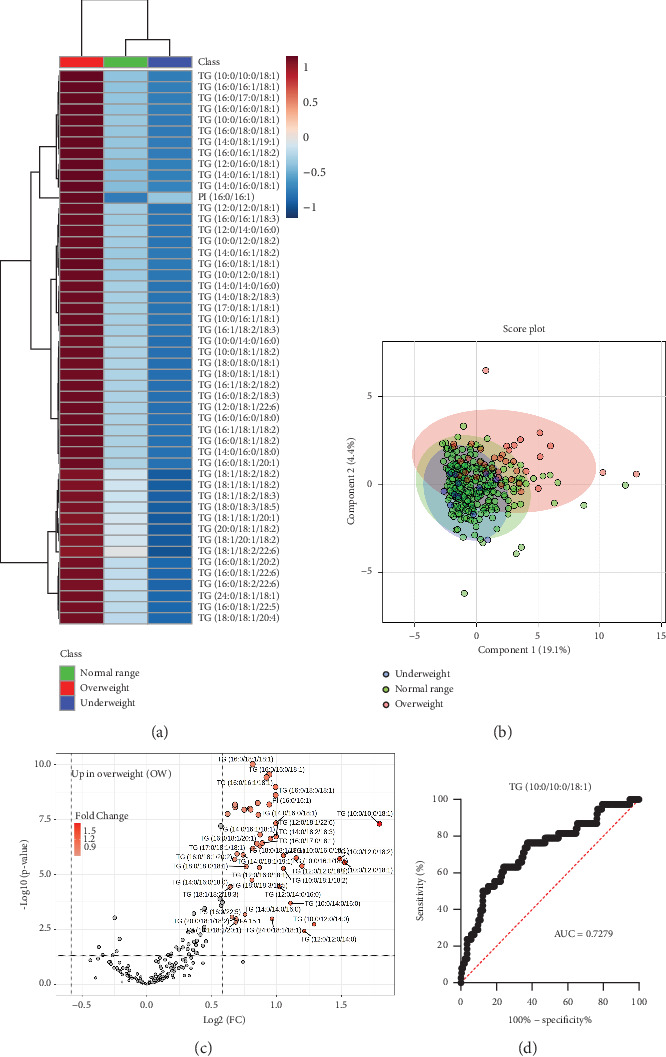
Variation in underweight (UW), normal range (NR), and overweight (OW) groups of preadolescent children's plasma lipidome. (a) Hierarchical cluster correlation analysis of the Top 50 significantly altered lipids (*p* < 0.05) in UW, NR, and OW groups. The intense red color indicates high-concentration lipids, whereas the intense blue color indicates low-concentration lipids in the respective groups. (b) Multivariate analysis of sparse partial least squares discriminant analysis (sPLS-DA) score plot of UW, NR, and OW groups. (c) Volcanic plot representing significantly altered lipids (*t*-test, *p* < 0.05) in the plasma samples of groups of NR versus OW children. (d) Receiver operating characteristic (ROC) analysis of significantly altered TG (10:0/10:0/18:1) lipid which gave the highest area under the curve (AUC) value.

The POW is based on age- and sex-specific standard body weight for height and is used to define childhood obesity in Japan [31]. POW was calculated as [measured weight (kg) − standard weight (kg)/standard weight (kg)] × 100. Children with POW ≤ −20%, POW − 20% ~ +20%, and children with POW ≥ +20% were classified as underweight (UW), normal range (NR), and overweight (OW), respectively (Table 2). The cluster correlation analysis results of the Top 50 significantly altered lipids between the UW, NR, and OW groups are shown in Figure 5(a). Compared with UW and NR groups, most of the TG was increased in the OW groups. In particular, TG (10:0/10:0/18:1) showed a significant increase in the OW group.

In Figure 5(b), the multivariate sparse partial least squares discriminant analysis (sPLS-DA) score plot illustrates the distribution of annotated lipids among the different weight groups. Components 1 and Component 2, accounting for 23.5% of the total variance and, with Component 1 explaining 19.1%, depict the separation of sample groups based on their lipid profiles. Notably, the UW and NR groups exhibited overlapping clusters, suggesting minimal lipid compositional differences between these two weight categories. In contrast, the OW group samples form distinct clusters, indicating significant differences in lipidomic profiles between the UW and NR groups.  Figure 5(c) exhibits volcanic plots for the plasma lipidome of the NR and OW children, which describe significantly and largely altered lipid molecular species in terms of −log_10_ (*p* value) versus log_2_ (fold change) values. The results clearly show that most of the TG, especially capric acid chain-composed TG (e.g., TG (10:0/10:0/18:1)), were upregulated in the OW group. Receiver operating characteristic (ROC) curve analysis was performed to evaluate the diagnostic potential of this lipid in distinguishing OW from NR. Among all discriminatory lipids, TG (10:0/10:0/18:1) had the highest AUC (area under the curve) of 0.7279 (95% confidence interval (CI): 0.6377–0.8181), respectively (Figure 5(d)).

## 4. Discussion

Human blood is a vital biomarker that is extensively used in both research and clinical fields [32]. Blood plasma is a self-regenerating, well-defined biological fluid that can be easily collected with minimal health risks [33, 34]. Recently, there has been a notable emergence of MS-based lipidomics techniques, due to significant technological platforms for identifying hundreds to thousands of lipids in plasma [35] Before examining pathological alterations in the plasma lipidome, it is essential to comprehend the natural variability of the lipidome in healthy individuals and within normal parameters, considering anthropometric factors like age, gender, and weight. Studies have shown that sex, age, and weight exert significant effects on the longitudinal patterns of plasma lipids [36]. The results of this study are visualized in a lipid biosynthesis pathway as depicted in Supporting Information 1: Figure S1. Acetyl coenzyme A (acetyl CoA) plays a central role as a precursor molecule in the synthesis of various lipid classes. Acetyl CoA contributes to the formation of FAs, which are fundamental building blocks in lipid biosynthesis along with CE which belongs to the ST subclass of lipids. Acetyl CoA, in combination with dihydroxyacetone phosphate (DHAP), and glycerol-3-phosphate (G3P), initiates the production of lysophosphatidic acid (LPA), LPA which subsequently forms phosphatidic acid (PA), diacylglycerol (DAG), and TG. PA can further interact with cytidine diphosphate diacylglycerol (CDP-DAG) to generate phospholipids (PLs) and further GPs. Additionally, acetyl CoA can be converted into palmitoyl CoA. Palmitoyl CoA, in combination with the amino acid serine (Ser), leads to the formation of 3-dehydro-sphinganine and dihydrosphingosine. These intermediates are then used to produce Cer, which are key components of the SP class. Further, Cer helps in the synthesis of HexCer and SM.

SFAs play regulating roles in protein activation, protein–membrane interaction, and gene transcription, and hence in metabolism and biological functions [37]. As depicted in Figure 3, the SFA levels did not exhibit any significant differences between boy and girl children. However, when stratified by age groups, a noteworthy pattern emerged. SFAs were significantly elevated in the 9-year-old age group for, both boy and girl cohorts when compared with other age groups. Conversely, SFA levels displayed a decreasing trend with increasing age across both boy and girl children. Several studies show that free fatty acids (FFAs) have been significantly increased in the plasma samples of younger age (newborn) groups and female participants [38–40]. In a previous study involving newborns, infants, children, and young adults, plasma samples revealed elevated levels of SFA levels in younger age groups compared with other age groups [38]. Our findings from plasma samples of preadolescent children align with these observations. Furthermore, in line with a previous study on serum samples, no disparity in SFA levels was observed between boy and girl participants [41]. Elevated SFA levels in younger individuals compared with older individuals can be attributed to factors such as dietary habits, metabolic differences, hormonal influences during growth and development, and lifestyle factors like physical activity levels [42]. Collectively, these factors may contribute to higher plasma SFA levels among younger individuals. MUFAs improve cholesterol levels, reduce inflammation, regulate blood sugar, aid in weight management, and enhance fat-soluble vitamin absorption, whereas PUFAs improve cognition, mood regulation, TG, blood pressure, blood clot prevention, and eye and skin health [43]. The levels of total MUFAs and PUFAs, including *ω*-3 and *ω*-6 PUFAs, did not differ between the boy and girl groups in our study, nor did they differ by age groups among children (Figure 3). The present findings align with earlier research on total PUFA levels in the blood samples from 160,000 individuals aged 10–99 years [44]. Consistent FA profiles across demographic categories can be attributed to similarities in physiology, comparable hormonal effects, stable dietary patterns, genetic influences, and methodological factors affecting FA metabolism and distribution [45]. The reason for this is unknown, but several elements could contribute to FA composition consistency in previous studies. Furthermore, compared with blood samples from adults and newborns, the total MUFAs in children's samples were significantly lower [46].

GPs are primary constituents of cell membranes and act as precursors for signaling molecules involved in numerous cellular and physiological processes [6]. Plasmalogens have antioxidant activity, thereby exhibiting protective effects on cells, plasma, and tissues by mitigating oxidative stress [47]. Most lipid classes exhibited some level of differentiation across all age groups between boys and girls (Figure 4). PEs are the second most abundant class of PLs in cells and biofluids [48]. The results of the present study showed that PE species contributed to the differentiation between different age groups in both boy's and girl's plasma samples. In our findings, PE lipid molecular species increased in the 11-year-old age group, whereas PS lipid molecular species increased in the 12-year-old age group compared to the 9-year-old age group in both sexes of plasma samples. In particular, PE (O-17:1/20:4) and PE (O-18:2/20:5) exhibited patterns consistent with prior investigations of blood samples from children aged 0–13 years [21]. An earlier examination of serum samples from healthy individuals noted increased levels of certain molecular species of PE between elderly and young Japanese individuals of both genders [41]. In addition, a recent study highlighted a sex-dependent, age-related rise in PLs, including PCs and PEs, specifically in females rather than males. This finding could be attributed to various physiological and developmental factors, including changes in hormone levels, alterations in dietary habits, metabolic shifts associated with growth and development, and other biological processes occurring during puberty [39]. Additionally, genetic and environmental factors may also influence the specific lipid profiles observed across different age groups [49].

SPs are structural components of the cell membrane and play a role as a cellular messenger [50]. Interestingly, low levels of specific SM molecular species are linked to an increased risk of Type 2 diabetes (T2D), cardiovascular disease, and neurodegenerative disease [51, 52]. Moreover, age exhibited a robust correlation with Cer species and HexCer species, which are derived from an atypical de novo SP synthesis pathway [3]. Cer and HexCer ensure the integrity, functionality, and adaptation of cell membranes, which are crucial for overall cellular and tissue health [53]. In our study, Cer, SM, and HexCer levels were elevated in the 10-year-old age group of boy and girl plasma samples (Figure 4). A prior study noted higher SM levels in elder females than in elder males, although levels were similar between young and elderly females. Additionally, it was reported that young children with low sex hormone levels displayed sex-related differences in plasma SM levels. However, Cer species exhibit age-related increases in serum samples among male subjects [54]. Moreover, specific SM molecular species, such as SM 32:1, are reduced in individuals with T2D [51].

TG-containing SFAs undergo *β*-oxidation in the mitochondria, and aging induces impaired activity of *β*-oxidation enzymes [55]. A recent investigation revealed that increased levels of specific TG molecular species are associated with an increased risk of T2D [41, 56]. Additionally, it noted higher serum levels of these TG molecular species in elderly individuals compared to younger individuals, which is consistent with our results. This study further revealed that plasma levels of most TG, particularly TG (10:0/10:0/18:1) were higher in boys than in girls. However, elucidating the role of lipid species in obesity-related metabolic changes remains challenging [57]. The samples were grouped based on their POW distribution. In this investigation, the majority of TG lipid subclass species (e.g., TG (10:0/10:0/18:1)) exhibited increased levels in the OW group compared to both the NR and UW groups (Figure 5), which is consistent with earlier research that links various TG to OW [58]. Furthermore, all distinct TG demonstrate a notable association with weight.

The selection of fasting or nonfasting samples for lipid measurements depends on the clinical scenarios. For example, for the diagnosis of metabolic syndrome and estimating the initial risks in an untreated primary prevention patient, the nonfasting samples were preferred over fasting samples. Whereas to screen the patients having a family history of genetic hyperlipidemia, fasting samples were required [59]. Nonfasting lipid measurements have several advantages over fasting as they simplify blood sampling for patients and clinicians and improve patient compliance with lipid testing. A study by Nordestgaard et al. demonstrated that fasting is not routinely required for the determination of lipid profile, and they recommended the use of nonfasting blood samples for the assessment of plasma lipid profile [60]. Another study by de Vries, Klop, and Castro Cabezas showed that nonfasting lipid profile is acceptable for the determination of lipid-lowering therapy, cardiovascular risk assessment, and for the discrepancy between primary and lipid disorders [61]. A prospective study involving 26,509 healthy women in the United States demonstrated that nonfasting blood TG was associated with cardiovascular events rather than fasting TG levels [62]. A study by Szternel et al. established a reference value for nonfasting lipid parameters in healthy 9–11-year-old children and demonstrated the significance of nonfasting blood lipid parameters in pediatric cardiovascular risk assessments [63]. Another study involving 356 French–Canadian pediatric population aged 6–13 years old also established the reference intervals for lipid markers based on age, sex, and puberty stage in nonfasting plasma samples to screen [64]. All these research evidences suggest that obtaining the nonfasting blood lipid levels is also very important as compared to fasting samples. Hence, despite the small sample number, we obtained the nonfasting plasma lipid profiles of children as a first report on a Japanese pediatric population-based study.

This study contributes to the identification of diverse lipid classes in plasma samples from preadolescent children, categorized by sex, age, and weight groups, with a thorough analysis of lipid composition. However, several limitations should be addressed in future research. These limitations include the semiquantitative nature of the reported concentrations rather than the absolute levels. Nonfasting plasma samples were collected from preadolescent children and analyzed. Because the lipid composition may vary depending on factors such as diet, physical activity, genetic factors, and family history, which are not considered in this study. In addition, although various molecular species of SPs, GLs, and FAs were detected in plasma samples from children in both boys and girls, the precise mechanisms underlying their metabolism remain unclear. Apart from these limitations, this study offers a comprehensive lipid profiling of preadolescent children's plasma and their variations with sex, age, and POW groups. The key strength of this study was that we applied the untargeted LC/MS technique and determined capric acid-containing TG could be a plausible plasma biomarker for childhood obesity. However, the clinical significance and reproducibility of these findings need further validation.

## 5. Conclusions

In this study, plasma samples from preadolescent children were compared among different sexes, ages, and weight categories using lipid profiles. The analysis identified distinct relative concentrations of lipid species within the 9–12-year-old age group for both boys and girls. The statistical analysis revealed significant changes in SFA levels between 9-year-old children and different age groups of both sexes. SPs, including Cer, HexCer, SM, and PL such as PI, exhibited relatively higher concentrations in the plasma of individuals within the 10-year-old age group compared with other age cohorts. Additionally, plasma TG and PS levels increased within the age range of 9–12 years. Furthermore, OW children showed upregulated TG compared to NR participants, with TG (10:0/10:0/18:1) as a potential marker for childhood obesity. In conclusion, this study highlighted significant variations in the lipidome, demonstrating the effectiveness of untargeted lipid profiling using HPLC/LTQ-MS as an analytical tool for investigating the global lipid profile in preadolescent plasma samples. This research offers valuable insights into sex, age, and weight-specific observations of lipid metabolism in preadolescent children.

## Figures and Tables

**Table 1:** Characteristics of participants in the Hokkaido study (mean ± SD).

	**Age (years old)**	**Height (cm)**	**Weight (kg)**	**POW (%)**
Boys (*n* = 181)	9 (*n* = 68)	134.9 ± 5.0	31.8 ± 6.1	1.40 ± 13.56
	10 (*n* = 63)	139.0 ± 6.4	35.0 ± 8.3	2.31 ± 16.90
	11 (*n* = 20)	148.3 ± 6.5	41.8 ± 8.1	2.69 ± 18.54
	12 (*n* = 30)	150.6 ± 9.4	44.2 ± 9.8	5.12 ± 17.81

Girls (*n* = 161)	9 (*n* = 46)	136.1 ± 5.7	30.2 ± 5.9	−5.18 ± 11.72
	10 (*n* = 70)	140.4 ± 7.0	34.6 ± 8.1	0.07 ± 14.04
	11 (*n* = 16)	151.0 ± 5.0	39.5 ± 5.8	−6.83 ± 10.05
	12 (*n* = 29)	151.5 ± 6.5	42.6 ± 8.4	−2.75 ± 12.69

**Table 2:** Number of children categorized by POW (%).

**Age (years old)**	**Boys (** **n** = 181**)**			**Girls (** **n** = 161**)**		
	**POW (%)**			**POW (%)**		
	**Underweight**	**Normal range**	**Overweight**	**Underweight**	**Normal range**	**Overweight**
9	2	57	9	3	42	1
10	0	53	10	3	60	7
11	0	16	4	2	14	0
12	1	24	5	1	26	2

*Note:* Underweight: children with POW < −20%; normal range: children with POW between −20% and 20%; overweight: children with POW ≥ +20%.

Abbreviation: POW, percentage of overweight.

## Data Availability

The data that support the findings of this study are available from the corresponding authors upon reasonable request.

## Supporting Information

Supporting Information 1 Figure S1: Analysis of lipid biosynthesis pathways in boys and girls. The data is represented as mean ± SEM. The *y*-axis represents the concentration of the lipid subclass in nanograms per milliliter, and the *x*-axis represents different sex groups, where the abbreviations are as follows: acetyl coenzyme A (acetyl Co A), fatty acids (FAs), dihydroxyacetone phosphate (DHAP), glycerol-3-phosphate (G3P), lysophosphatidic acid (LPA), phosphatidic acid (PA), cytidine diphosphate diacylglycerol (CDP-DAG), phosphatidylinositol (PI), phosphatidylethanolamine (PE), lyso-phosphatidylethanolamine (LPE), phosphatidylcholine (PC), lysophosphatidylcholine (LPC), phosphatidylserine (PS), diacylglycerol (DAG), triacylglycerol (TG), serine (Ser), ceramide (Cer), hexosylceramide (HexCer), and sphingomyelin (SM). Figure S2: Volcanic plot representing significantly altered lipids (*t*-test, *p* < 0.05) in the plasma samples of boys: (A) boys in the 9 versus 10 years age group. (B) Boys in the 9 versus 11 group. (C) Boys in the 9 versus 12 group. (D) Boys in the 10 versus 11 group. (E) Boys in the 10 versus 12 group. (F) Boys in the 11 versus 12 group. Figure S3: Volcanic plot representing significantly altered lipids (*t*-test, *p* < 0.05) in the plasma samples of girls. (A) Girls in the 9 versus 10 group. (B) Girls in the 9 vs. 11 group. (C) Girls in the 9 versus 12 group. (D) Girls in the 10 versus 11 group. (E) Girls in the 10 versus 12 group. (F) Girls in the 11 versus 12 group.

Supporting Information 2 The study is aimed at profiling the plasma lipidome of preadolescent children aged 9–12 years living in Hokkaido, Japan, and its variations with sex, age, and body weight using untargeted liquid chromatography/mass spectrometry.
