# Supplementary figures and images for: Plasma Lipidomics of Preadolescent Children: A Hokkaido Study
Source: J Lipids. 2025 Feb 16;2025:3106145. doi: 10.1155/jl/3106145 (PMC11898111; doi:10.1155/jl/3106145)

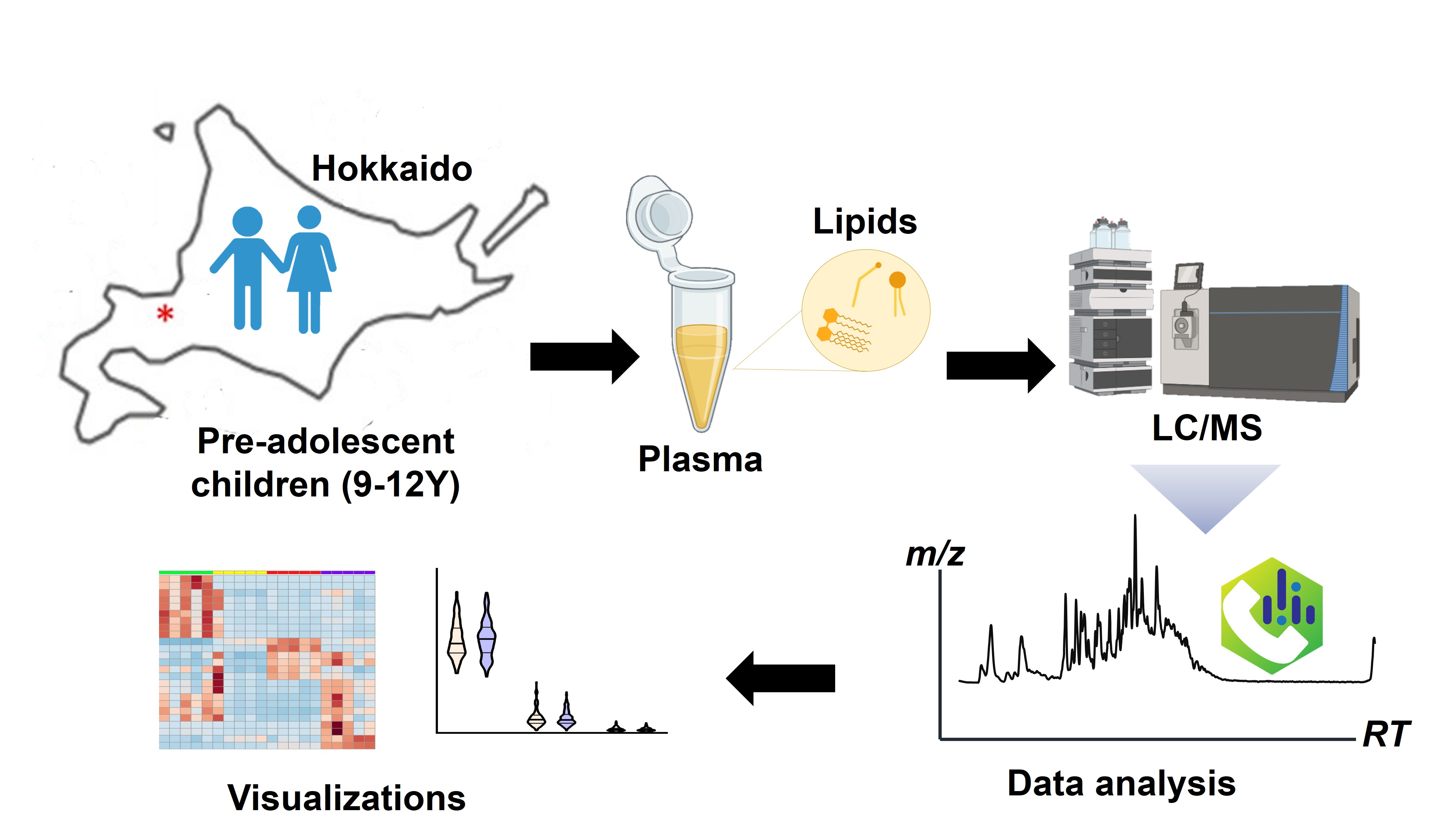

Supplement: Supporting Information 2 — The study is aimed at profiling the plasma lipidome of preadolescent children aged 9–12 years living in Hokkaido, Japan, and its variations with sex, age, and body weight using untargeted liquid chromatography/mass spectrometry. [file 3106145.f2.jpg]
